# P-400. Surgical Site Infections among Heart Transplant, Lung Transplant, and VAD Inserted Patients

**DOI:** 10.1093/ofid/ofae631.601

**Published:** 2025-01-29

**Authors:** Bipasha Choudhury, Stephanie W Smith, Maureen Buchanan-Chell

**Affiliations:** University of Alberta, Edmonton, Alberta, Canada; University of Alberta, Edmonton, Alberta, Canada; Alberta Health Services, Edmonton, Alberta, Canada

## Abstract

**Background:**

Surgical site infections (SSI) after heart and lung transplantation and implantation of Ventricular Assist Devices (VAD) confer significant morbidity. Despite this, many sites do not perform routine surveillance of this population.

Considering the paucity of available data, this retrospective chart review evaluated the incidence, risk factors, associated microorganisms, and outcomes associated with surgical site infection by type of transplant received.

**Methods:**

This review was conducted at a tertiary care hospital in Edmonton, Alberta. All patients who received a heart or lung transplant or VAD insertion between July 1, 2022, and December 31, 2023, that had at least 90 days of follow-up were included. Information collected included data on transplant/VAD insertion, age, sex, presence of SSI, microorganisms associated with infection, length of stay, and outcome. Other data collected included receipt of antibiotic prophylaxis, admission to hospital in the six months prior to transplant, and surgery in the six months prior to transplant.

**Results:**

A total of 219 patients were included in the review (double lung transplant = 104, heart transplant = 38 and VAD insertion = 77)

SSI rate was found to be higher in the VAD insertion group (16%), compared to lung (12.5%) or heart transplant (7.8%) recipients. Post-transplant hospital stay was longer in lung transplant (17-131; median 55) and heart transplant recipients (81-115; median 112) who developed SSI, compared to their counterparts who did not develop SSI (12-197; median 32 and 13-120; median 20 respectively).

Coagulase-negative staphylococcus aureus was found to be the most common pathogen among all three groups of transplants that developed surgical site infections (SSIs). However, *Cutibacterium acne* was also frequently isolated in VAD recipients, while Yeast, *E. fecalis* and *E. feceium* were commonly found to be responsible for lung transplant SSIs.

**Conclusion:**

Rates of SSI after heart and lung transplantation and VAD insertion are high and result in significant morbidity. The choice of antibiotic prophylaxis may need to be individualized in this patient population given prior antibiotic exposure, hospitalization, and risk of more resistant pathogens.

**Disclosures:**

**All Authors**: No reported disclosures

